# Obstructive Sleep Apnea in Pregnancy and its Impact on Maternal-Fetal Health: A Hidden Threat - Narrative Review

**DOI:** 10.2174/0118743064411633250826105304

**Published:** 2025-08-29

**Authors:** Parth Dhamelia, Vanshika Gupta, Srishty Agarwal, Baltej Singh, Rohit Jain

**Affiliations:** 1 Maulana Azad Medical College,, New Delhi- 110002, India; 2 Government Medical College, Surat -395001, India; 3 Department of Internal Medicine, Christiana Hospital, Newark, Delaware, USA; 4 Department of Internal Medicine, Penn State Milton S. Hershey Medical Center, Hershey, United States

**Keywords:** Obstructive sleep apnea, Intrauterine growth retardation, Body mass index, Progesterone, Hypertensive disorder of pregnancy, Polysomnography

## Abstract

Obstructive sleep apnea (OSA), characterised by apnea or hypopnea, often presents with symptoms such as gasping or snoring. However, these symptoms can be nonspecific and are frequently overlooked, particularly in pregnant women, where they are often attributed to normal physiological adaptations, leading to underdiagnosis and negative maternal and fetal outcomes. This narrative review examines the implications of OSA during pregnancy, highlighting the importance of early screening and evaluating available treatment options. We reviewed various articles on PubMed and Google Scholar about the impact of OSA during pregnancy, screening methodologies, and treatment effectiveness. OSA often increases sympathetic activity along with immune dysfunction, resulting in adverse outcomes like gestational hypertension, preeclampsia, gestational diabetes, cardiomyopathy, depression, and higher rates of cesarean deliveries, while the fetus suffers from intrauterine growth restriction (IUGR), preterm births, and perinatal mortality. Various screening tools, such as the Berlin Questionnaire, Epworth Sleepiness Scale (ESS), STOP-BANG, and Wisconsin questionnaires, aid in early diagnosis. Treatment options include lifestyle modifications, positive airway pressure (PAP) therapy, either continuous (CPAP) or bilevel (BiPAP), hypoglossal nerve stimulation (HGNS), mandibular advancement devices (MAD), and maxillomandibular advancement (MMA) surgery, with CPAP being identified as the preferred treatment. To reduce adverse outcomes for both the mother and the fetus, early detection and treatment of OSA in pregnant women are essential. Increased awareness among expectant mothers, routine screening using validated questionnaires, and appropriate treatment selection can not only decrease fetal complications but also reduce the risk of long-term adverse effects of OSA on maternal health.

## 1 INTRODUCTION

Obstructive Sleep Apnea (OSA), caused by inspiratory upper airway collapse during sleep, is defined as five or more episodes of apnea (*i.e*., cessation of airflow for 10 seconds or more) and/or hypopnea (*i.e*., reduction in airflow by at least 30% for 10 seconds or more) per hour of sleep, resulting in a ≥3% drop in oxygen saturation (normal SpO_2_ ≥95%), hypercapnia (rise in pCO_2_), and arousal from sleep. It is accompanied by nocturnal breathing disturbance symptoms, such as snoring, snorting, gasping, or breathing pauses during sleep, which can lead to daytime fatigue and sleepiness [1]. Global estimates indicate that approximately 936 million adults between the ages of 30 and 69 have obstructive sleep apnea (OSA), of whom 425 million adults have moderate to severe OSA [2]. The National Healthy Sleep Awareness Project estimates that over 25 million adults in the United States suffer from OSA, with prevalence rates increasing significantly over the past 20 years, most likely due to the obesity pandemic, which affects approximately 26% of adults aged 30 to 70 years [3]. Prevalence is even higher among middle-aged (44–64 years) and elderly populations (≥65 years), with one study indicating a significant likelihood of developing obstructive sleep apnea in 56% of individuals aged 65 years and above [4]. The American Academy of Sleep Medicine (AASM) reports that OSA symptoms affect 24% of males and 9% of females [5]. The higher prevalence in men is attributed to factors such as longer upper airway length due to sex hormones, central distribution of adipose tissue, and higher visceral fat levels compared to females [6]. Desaturation following longer apneic episodes (>30s) could be more detrimental in females than in males, and since males and females exhibit different clinical presentations, females with OSA often remain underdiagnosed compared to males [7]. They are more prone to experience nonspecific symptoms like sleeplessness, mood swings, nightmares, exhaustion, and low energy, and report a higher frequency of sick days [8]. Pregnant women are more likely than other women of reproductive age to experience sleep disturbances resul-ting from physiological changes during pregnancy, such as pharyngeal narrowing and nasal obstruction, making them particularly vulnerable. Moreover, pregnancy-related edema and weight gain can exacerbate upper airway collapsibility, and hormonal changes may affect muscle tone and respiratory drive [9]. Pregnant women may present either with chronic OSA, which worsens during pregnancy, or gestational OSA, which develops due to weight gain and airway or respiratory changes, with the latter potentially improving or resolving completely after delivery [10, 11]. A high body mass index (BMI) at the start of pregnancy and increased edema in late pregnancy are associated with heightened snoring, with its frequency more than doubling between the first and third trimesters [12]. Pregnant women having a BMI over 35 kg/m2 are at a higher risk, while those with preeclampsia experience a more severe reduction in pharyngeal size; however, making adjustments to lifestyle and diet can help minimize the likelihood of OSA and its complications, especially when other conditions such as advanced age or chronic hypertension are present [13]. It was hypothesized that OSA may impact placental function through the above-mentioned potential mechanisms, a theory supported by evidence of placental tissue hypoxia and altered levels of placental secreted markers in women with OSA [14, 15]. This impact on the fetal placenta could result in fetal hypoxia, causing fetal growth restriction, preterm birth, and low birth weight. It has also been reported that women with OSA have a greater risk of premature delivery and rupture of membranes as compared to healthy women [16]. Intermittent hypoxia and disturbed sleep caused by OSA can cause hormonal and neurochemical imbalances, increasing sensitivity to mood disorders like depression and heightening stress reactions, which, in the case of perinatal depression, place an immense burden on both the developing fetus and the mother [17]. This review aims to analyze the sensitivity of pregnant females to OSA, the mechanisms involved in its development, its impact on pregnancy-related disorders, adverse fetal outcomes, and the psychological changes in the mother caused by OSA, as well as several screening and treatment alternatives to enhance health outcomes for both mother and child.

## 2 METHODOLOGY

We searched and reviewed various articles on PubMed and Google Scholar discussing the effects of OSA in preg-nancy and its maternal and fetal complications. Articles that were reviewed were not only based on OSA-related comp-lications but also on the diagnosis and treatment options available for pregnant females. The search included articles published from 2000 to 2024. The keywords used for the search included- Obstructive Sleep Apnea AND pregnancy, Maternal-fetal Outcomes AND sleep Apnea, OSA AND Gestational Hypertension, OSA AND Gestational Diabetes, Diagnosis of OSA AND pregnancy, Treatment of OSA AND pregnancy.

### 2.1 Inclusion and Exclusion Criteria

Studies were included if they:

1) Examined OSA during pregnancy and its impact on fetal outcomes.

2) Provided data on maternal complications like dia-betes, preeclampsia, premature deliveries, *etc*., fetal compli-cations like low birth weight, congenital anomalies, *etc*.

Studies were excluded if they:

1) Focused more on the non-pregnant population or discussed sleep disorders other than OSA

2) Did not provide sufficient clinical evidence and data related to maternal-fetal implications

3) Not published in English

## 3 PATHOPHYSIOLOGY

Obstructive sleep apnea (OSA) develops as a result of the interaction of several factors. Besides the anatomy of the upper airway that makes it susceptible to collapse, several non-anatomical factors contribute to the development of OSA, some of which are exacerbated by physiological changes that occur during pregnancy.

The overall effects of OSA in pregnancy are summarized in Fig. (**1**).

### 3.1 Physiological Changes in Pregnancy

Pregnancy triggers numerous physiological changes, most of which are driven by hormones secreted by the placenta to support the developing embryo. These hor-mones include elevated levels of human chorionic gona-dotropin (hCG), human placental lactogen (hPL), proges-terone, estrogen, and cortisol [18, 19]. Progesterone acts as a respiratory stimulant, increasing respiratory drive and ventilation, enhancing the sensitivity of upper airway dilator muscles during sleep, and preventing carbon dioxide accumulation [20]. Pregnant women with lower progesterone levels are more likely to develop OSA, suggesting that adequate progesterone may help prevent its onset [21]. In contrast to progesterone, the mechanism by which estrogen contributes to the development of OSA remains unknown. One study has indicated that nasal patency decreases during pregnancy due to vasodilation and hyperemia caused by the activation of estrogen receptors in the nasal mucosa, which may contribute to upper airway obstruction [22, 23]. However, this contrasts with a recent study where using nasal dilator strips to relieve the supposed nasal upper airway obstruction did not affect apnea/hypopnea events in pregnant females [24]. Along with hormonal changes, specific anatomical changes also occur during pregnancy, which can lead to OSA. Pregnancy can lead to the development of soft tissue edema due to increased fluid retention and fat deposition in soft tissues, which may contribute to sleep apnea during pregnancy. In this condition, the airway may collapse under the edematous soft tissue in the recumbent position [25]. The enlarging gravid uterus during pregnancy pushes the diaphragm upward, increasing abdominal pressure, especially during the third trimester, which induces basal atelectasis [26] and insufficient alveolar ventilation, leading to CO_2_ retention and exacerbating the effects of obstructive sleep apnea. Finally, systemic inflammation mediated by multiple cytokines and immuno-modulators that increase during pregnancy can lead to airway swelling and inflammation, thereby increasing the likelihood of developing obstructive sleep apnea [27].

### 3.2 Maternal Pathophysiology in OSA

**Fig. (2):**
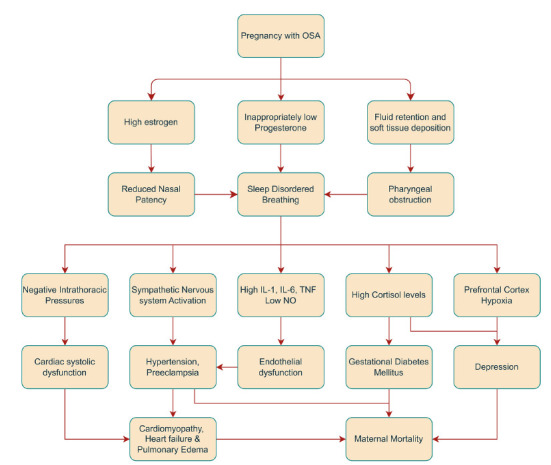
Pathophysiological pathway for effects of OSA on maternal health.

Obstructive sleep apnea causes widespread effects on maternal health during pregnancy. Sleep-disordered brea-thing leads to the development of various maternal hyper-tensive disorders, including gestational hypertension, pre-eclampsia, and eclampsia [28]. Sleep-disordered breathing causes widespread inflammation induced by reactive oxygen species (ROS) and inflammatory molecules generated downstream of the nuclear factor kappa B (NFKB) pathway, such as tissue necrosis factor-alpha (TNF-alpha) and interleukin-6 (IL-6) [29]. This, along with low nitric oxide (NO) levels, contributes to widespread endothelial dysfunction and causes hypertension [30]. Continuous positive airway pressure (CPAP) treatment decreases the risk of preeclampsia, which prevents upper airway collapse, targeting the anatomical factors that cause OSA. However, the risk does not reach the same level as in pregnant women without sleep apnea [31]. Non-anatomical variables may contribute to the development of OSA and hypertension during pregnancy, which CPAP therapy does not address. Apnea syndromes in pregnancy are also linked to the development of gestational diabetes mellitus (GDM). This is most likely caused by sympathetic nervous system activation-induced hepatic insulin resistance, as indicated by elevated normetanephrine and inflammatory markers, such as interleukin-1 (IL-1) [32], in females with low oxygen saturation. This pathogenetic mechanism may be supported by the fact that CPAP therapy, which reduces sympathetic activation related to upper airway collapse-induced hypoxia, also improves insulin resistance [33]. Obstructive sleep apnea causes frequent nighttime waking, resulting in fragmented sleep and elevated cortisol levels. This further perpetuates the rise in glucose levels by increasing insulin resistance and stimulating the production of glucose [34]. Pregnancies with OSA are also associated with cardiovascular dys-function and pulmonary edema [35]. OSA is associated with more negative intrathoracic pressures than usual, as the thoracic inspiratory muscles exert greater effort to overcome the upper airway obstruction. These negative pressures may be transmitted to the heart, preventing it from contracting efficiently (systolic dysfunction) [36]. Additionally, hypertension associated with OSA contri-butes to further systolic dysfunction, as the heart must pump against the higher pressure of the blood column (*i.e*., increased afterload). Moreover, diabetes mellitus resulting from the above-mentioned mechanisms can cause an inappropriately elevated renin-angiotensin-aldosterone system and increased oxidative stress, leading to the development of dilated cardiomyopathy [37]. Cardiac dysfunction arising as a result of these mecha-nisms, combined with the high circulating blood volume (due to fluid retention during pregnancy), contributes to pulmonary vascular congestion and subsequently an increased hydrostatic pressure, leading to pulmonary edema [38]. Pulmonary edema may also result from endothelial dysfunction caused by widespread inflam-mation, allowing for easier fluid extravasation. These comorbidities naturally lead to higher rates of cesarean deliveries, both urgent and planned, and account for the longer duration of hospital stays, increased ICU admis-sions, and elevated maternal mortality rates associated with obstructive sleep apnea in pregnancy [39, 40]. Obstructive sleep apnea is also linked to a variety of psychiatric disorders like depression, generalized anxiety disorder (GAD), and post-traumatic stress disorder (PTSD) in the peripartum period of pregnancy (Fig. **2**) [41, 42]. Certain studies indicate that these disorders result from hormonal imbalances, such as elevated cortisol, pro-gesterone, and estrogen levels during pregnancy [43, 44]. These psychological disturbances are also partly explained by preferential hypoxic damage to the prefrontal cortex in the frontal lobe, causing alterations in working memory and behavior [45], as well as persistent fatigue resulting from sleep disruption caused by apnea cycles [46].

### 3.3 Fetal Pathophysiology in OSA

**Fig. (3):**
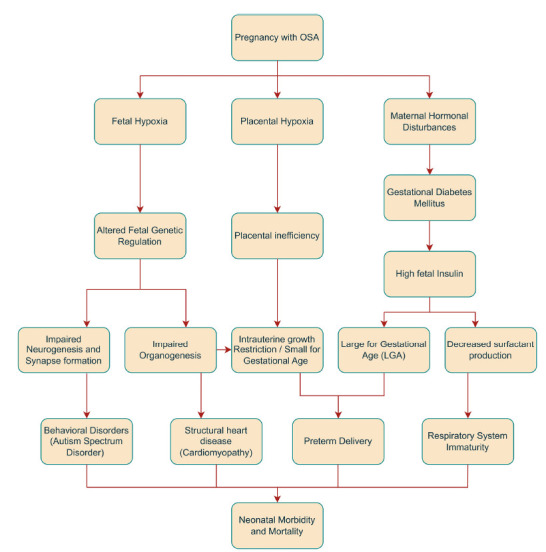
Pathophysiological pathway for the effects of OSA in pregnancy on fetal health.

Maternal hemodynamic adaptations are essential for maintaining gestation and fetal development. OSA during the first trimester has a substantial impact on the process of organogenesis, with a significant effect on neuronal development [47, 48]. There is mounting evidence inter-linking fetal hypoxia with epigenetic mechanism alter-ation; DNA methylation, histone modifications, and non-coding RNAs (ncRNAs), including long noncoding RNAs (lncRNAs) and microRNAs, are the principal mediators of epigenetic modifications, which lead to malfunctioning genes and signalling proteins, ultimately leading to deformities [49]. While uteroplacental insufficiency caused by placental hypoxia can lead to intrauterine growth retardation (IUGR), resulting in small-for-gestational-age (SGA) and low birth weight (LBW) newborns, studies have also found a comparatively higher risk of large-for-gestational-age (LGA) births in children born to mothers with OSA [50, 51]. This may be explained by the anabolic effects of elevated insulin and glucose levels in OSA mothers due to peripheral insulin resistance. Another anticipated outcome of OSA in pregnancy is an increased rate of preterm births. Possible reasons include low pro-gesterone levels seen in mothers with OSA, as pro-gesterone is needed to sustain pregnancy, and uterine overdistension caused by macrosomic babies due to the anabolic effects of gestational diabetes mellitus on fetal development [52]. Finally, intrauterine infections associated with gestational diabetes mellitus, poor respiratory maturity in preterm infants with subsequent neonatal respiratory distress (NRDS), and hypoxic-ischemic brain injury due to pro-longed labor in macrosomic neonates can explain the higher incidence of low Apgar scores in newborns, perinatal mortality, and stillbirths (Fig. **3**) [**39**].

## 4 DIAGNOSIS

Screening followed by appropriate treatment can improve sleep quality, thereby normalizing AHI and oxygen saturation levels and minimizing adverse health outcomes [53]. The severity of OSA can be determined using the apnea-hypopnea index (AHI), calculated by dividing the total number of apneas and/or hypopneas by the total sleep time. Based on this index, OSA is classified as mild (5–14 events per hour), moderate (15–30 events per hour), or severe (more than 30 events per hour). Other, less commonly used indices include the Respiratory Disturbance Index (RDI) and the Respiratory Event Index (REI). The RDI also accounts for respiratory effort-related arousals (RERAs) in addition to apneas and hypopneas. An RERA can be defined as an increased effort in breathing lasting 10 seconds or more due to airflow restriction, causing arousal before the criteria for apnea and hypopnea are met [54, 55].

The present screening questionnaires such as Epworth Sleepiness Scale (ESS), the STOP Questionnaire (Snoring, Tiredness, Observed Apnea, High Blood Pressure), the STOP-Bang Questionnaire (STOP Questionnaire plus BMI, Age, Neck Circumference, and Gender), the Berlin Ques-tionnaire, and the Wisconsin Sleep Questionnaire [56], were designed for the nonpregnant population, and research has shown limited sensitivity and specificity when applied to pregnant women, hence a new OSA scree-ning questionnaire is needed. Since pregnancy involves continual physiological changes, serial monitoring and screening for OSA are essential in each trimester [57]. The Berlin and ESS questionnaires are weak indicators of sleep apnea in the pregnant population due to their reliance on symptoms like fatigue and daytime sleepiness, which are extremely common during pregnancy, leading to false positives [58, 59].

Additionally, the Berlin questionnaire applies a binary BMI cutoff (≥30kg/m^2^) without accounting for the nature of weight changes during pregnancy [58]. A 2016 sys-tematic review reported pooled sensitivity and specificity for Berlin in pregnancy at 0.66 and 0.62, and for ESS at 0.44 and 0.62, indicating limited accuracy [60]. These overlapping symptoms often lead to under-recognition of sleep-disordered breathing as normal gestational changes. This highlights the need for developing pregnancy-specific screening tools.

Facco FL’s prospective study found that a simplified four-variable screening test, which incorporates self-reported frequent snoring, chronic hypertension, BMI, and age, predicts sleep apnea with high sensitivity and specificity [59]. In a prospective study, Facco FL dis-covered that a simple four-variable screening test, which includes snoring, chronic hypertension, BMI, and age, quite accurately predicts sleep apnea [59]. Another pro-spective study found that a model that takes into account age, BMI, and tongue enlargement (BATE) is more accurate in predicting the risk of OSA in African American women. However, the ESS, OSAHS risk, and SASS subscale of the MVAP-risk score showed lower predictive values when assessing OSA risk during pregnancy [61]. Overnight PSG investigations are tedious, costly, have limited availability, and are difficult to schedule for pregnant women, especially in late gestation. Home Sleep Testing (HST) is a reliable, easy, and affordable way for assessing high-risk individuals for OSA [62]. Home sleep tests measure various nighttime cardiac and respiratory parameters, such as respiratory rate, heart rate, oxygen saturation, heart rhythm, and blood pressure, to identify apneic episodes. Several HST devices have been proven in pregnant women [63, 64]. Pregnant women can easily utilize the Watch-PAT device, a wrist-worn diagnostic tool for SDB, which has a low failure rate. It can facilitate rapid screenings, reduce diagnostic waiting time, and enable early treatment, leading to better maternal and fetal outcomes [64].

## 5 TREATMENT

Pregnancy is a highly sensitive condition in which even minor disturbances in normal homeostasis can lead to severe complications. Obstructive sleep apnea (OSA) in pregnancy is associated with significant adverse outcomes for both the mother and fetus, including eclampsia, gestational diabetes, heart failure, fetal hypoxia, intra-uterine death, and increased maternal mortality. The reported prevalence of OSA in obese females can be as high as 43% during the second and third trimesters, with approximately one in five experiencing severe OSA [65]. This highlights the urgent need for active screening using questionnaires and early diagnosis through polysomno-graphy, home sleep testing (HST), or newer wearable technologies. Once diagnosed, prompt treatment is essen-tial to prevent OSA-related complications. However, the data related to guiding the treatment of OSA during pregnancy is insufficient. The studies attempting to assess the impact of treatment or safety during pregnancy were not adequately powered due to limited sample sizes. Instead, the benefits observed in the general population are extrapolated to pregnant women using existing treatment standards [66]. The ethical implications of assigning an inferior treatment to a vulnerable population, such as pregnant women, may explain the lack of randomized clinical trials supporting one treatment over another.

Addressing the risk factors for OSA through lifestyle modifications is a critical aspect of management. Obesity, the most significant risk factor, can be mitigated through weight control strategies, including dietary changes and medical therapies. These interventions have been shown to reduce symptom burden and lower mortality from cardiovascular events such as myocardial infarctions and strokes in OSA patients [67, 68]. Preventive measures, such as positional therapy, have been associated with a reduction in the severity of OSA in pregnant women. In cases where CPAP is not readily available or practical, these measures may be considered [69]. Lifestyle modifi-cations, such as avoiding supine positioning, may be recommended. Left lateral decubitus positioning during sleep is particularly effective in reducing position-related apneic events, especially in obese females [70]. Bariatric surgery is often employed for OSA patients with severe obesity, demonstrating superior efficacy compared to lifestyle modifications alone, with more than 60% of patients achieving OSA remission [71]. However, in pregnant females, the potential benefits of OSA remission post-bariatric surgery must be weighed against the possible adverse maternal and fetal outcomes, necessi-tating further research in this area [72].

A preferred mode of treatment for OSA during preg-nancy is continuous positive airway pressure (CPAP) therapy [73]. CPAP delivers pressurized air through a mask to create a pneumatic splint that keeps the airway open throughout the respiratory cycle, preventing apneic and hypopneic episodes [74]. Studies have shown that CPAP reduces complications such as gestational hyper-tension and preeclampsia in pregnant females with OSA [73]. It has also been shown to reverse cardiomyopathy by improving heart rate and stroke volume, as well as enhancing insulin sensitivity [75]. However, CPAP did not significantly alter mean glucose levels in OSA patients with gestational diabetes [76]. CPAP has also been associated with decreased systemic inflammation, as indicated by reductions in markers such as interleukin-6, interleukin-8, C-reactive protein, uric acid, and tumor necrosis factor-alpha [75, 77]. Despite the known clinical benefits, the use of CPAP in pregnancy may be limited due to low compliance rates of around 33% as per a large randomized trial [55]. In another real-world cohort study, adequate compliance (use for ≥4 hours/night on 70% of nights) was achieved in only a minority of patients [31]. Therefore, more conclusive trials are necessary to eva-luate the effectiveness of CPAP in this patient population.

Bilevel positive airway pressure (BIPAP) is another option for positive airway pressure therapy. Unlike CPAP, BIPAP alternates between two levels of pressure, deli-vering a higher level during inspiration and a lower level during expiration, while allowing spontaneous breathing [78]. BIPAP serves as a viable second-line treatment for patients who fail or cannot adhere to CPAP therapy (defined as <4 hours of nightly use) or for those with co-existing conditions such as neuromuscular disorders, severe hypercapnia, or morbid obesity (BMI > 42.6 kg/m^2^) [79]. Studies have shown that BIPAP can reduce the AHI by 50% (from 15.2 to 7.4 events per hour) and improve arterial blood gas parameters [80]. However, due to higher costs and limited evidence of superior efficacy compared to CPAP in uncomplicated OSA cases, BIPAP remains a second-line option [81, 82].

Other, less common treatments include hypoglossal nerve stimulation (HGNS) devices, mandibular advance-ment devices (MADs), and upper airway surgeries such as mandibulomaxillary advancement (MMA) surgery. HGNS devices, which reduce the AHI by 15 events/hour in the long term, are emerging as a promising option for moderate-to-severe OSA in patients intolerant of CPAP, particularly individuals with a BMI ≤ 32 kg/m^2^ and a smaller neck circumference [83, 84]. However, their high upfront costs and limited evidence of efficacy in pregnancy are significant barriers [85]. MADs, which reposition the lower jaw to open the airway, are another second-line treatment for mild to moderate OSA. However, they are less effective than HGNS devices and may cause compli-cations such as dental malocclusion [86]. Both MADs and CPAP have been shown to reduce systemic inflammation and lower blood pressure and cardiovascular risk [87, 88].

Surgical options such as MMA surgery are highly effective for CPAP-nonresponsive patients, achieving an approximately 80% success rate in treating OSA in individuals with a BMI >30 kg/m^2^ [89, 90]. Despite their efficacy, these surgeries are invasive and costly. Additi-onally, evidence guiding OSA treatment in pregnancy remains limited. Most studies on OSA treatments during pregnancy lack sufficient power due to small sample sizes, and current standards largely extrapolate benefits observed in the general population [66].

## 6 DISCUSSION

Understanding the links between sleep disturbances and adverse pregnancy outcomes is crucial for compre-hending the broader implications of these findings. The following discussion explains the serious risks linked with OSA in expecting women, including fetal development and other pregnancy issues, using selected study data.

### 6.1 Effects on Maternal Health

Pregnancies with OSA have a greater risk of compli-cations such as preeclampsia, GDM, preterm and cesarean births, depression, and maternal death. (Table **1**). In a 2021 retrospective cohort analysis of U.S. hospital data, pregnant women suffering from OSA had a 2.2 times greater risk of having preeclampsia compared to those without [91]. A 2018 meta-analysis found an odds ratio (OR) of 2.35 for preeclampsia among pregnant females having OSA [28]. A 2024 study on Korean pregnant women showed a much higher association, with an OR of 13.1, likely reflecting limitations due to the smaller sample size [92]. OSA is also linked to reduced total sleep time and hormonal imbalances that contribute to GDM. Women with GDM exhibited shorter sleep durations (364 minutes) and a higher prevalence of OSA (73%) compared to those without diabetes (464 minutes of sleep and 27% OSA prevalence) [93]. In contrast, a study by Bublitz *et al*. revealed a lower OSA prevalence of 17% among women with GDM, potentially due to the use of polysomnography, which applies more stringent diagnostic criteria. This study also highlighted a blunted cortisol awakening response, indicative of hormonal dysregulation in OSA, but found no differences in diurnal cortisol variation between pregnant women with and without OSA [94].

Depression and preterm birth are also more common in pregnancies complicated by OSA. A 2022 meta-analysis found that OSA increased the odds of depression during pregnancy (OR: 3.98; 95% CI: 2.74–5.77) and preterm birth (OR: 1.95; 95% CI: 1.55–2.45) [42]. Another 2021 meta-analysis by Lu *et al*., based on a larger sample, esti-mated a lower but more precise OR for preterm delivery (1.3; 95% CI: 1.26–1.51) [51]. Additionally, a study in Peru reported that poor sleep quality and a greater risk of OSA, as assessed by the Pittsburgh Sleep Quality Index and Berlin Questionnaire, were associated with increased prevalence of antepartum depression (OR: 2.36; 95% CI: 1.57–3.56), generalized anxiety (OR: 2.02; 95% CI: 1.36–3.00), and PTSD (aOR: 2.14; 95% CI: 1.43–3.21) [41].

While data on the link between untreated OSA and conditions like peripartum cardiomyopathy and pulmonary embolism are limited, some studies highlight significant risks. A retrospective analysis of maternal discharges from the National Inpatient Sample (NIS) database (1998–2009) found that OSA increased the risk of cardiomyopathy (OR: 9.0; 95% CI: 7.5–10.9) and pulmonary embolism (OR: 4.5; 95% CI: 2.3–8.9) [95]. According to a recent cohort study, women with OSA had a five-fold higher risk of cardio-vascular morbidity, with cardiomyopathy and pulmonary edema prevalence of 1.1% compared to 0.2% in the non-OSA group [69]. However, cardiomyopathy as an outcome remains underreported, necessitating further research.

Finally, OSA has been associated with higher in-hospital mortality during pregnancy. In Louis *et al*.'s retro-spective study, pregnant women with OSA had five times the risk of in-hospital mortality (OR: 5; 95% CI: 2.4–11.5) compared to those without OSA [95]. However, a recent cohort analysis of multi-state inpatient databases in the U.S. found no significant increase in in-hospital mortality, likely due to underdiagnosis and small sample size, as concluded by the authors [96]. These conflicting findings underscore the urgent need for further investigation into the relationship between OSA and maternal outcomes.

### 6.2 Effects on the Fetus

Increased sympathetic drive, oxidative stress, impaired endothelial function, and activation of the inflammatory system can result from OSA-induced intermittent hypoxia and hypercapnia. Together, these factors may hinder fetal development by reducing placental oxygen and nutrient exchange [40]. Retrospective case-control research by Ravishankar S *et al*. found that women with OSA had higher rates of fetal normoblastemia and increased ex-pression of carbonic anhydrase IX (CAIX), a marker of tissue hypoxia, in their placentas compared to controls, suggesting persistent fetoplacental hypoxia [15]. This adverse intrauterine environment may also affect the expression of specific fetal genes. According to a recent study done using womb-cell samples (WCB) from women with OSA in REM, fetal gene expression is strongly impacted by maternal OSA. According to the mRNA expression profile results, WCB samples from women with OSA exhibited differential expression of 3,258 mRNAs compared to those from women without OSA. In the OSA group, 568 mRNAs were considerably downregulated, while 2690 mRNAs were significantly upregulated. Following maternal OSA, 13 candidate genes were iden-tified as potentially dysregulated in WCB cells. This included one downregulated mRNA, PRKAR1A, and twelve upregulated mRNAs: PFN1, UBA52, EGR1, STX4, MYC, JUNB, MAPKAP1, IGF2, CAT, MCL1, PPP1CB, and AKT2. These genes are essential for numerous physiological functions; therefore, dysregulation of these genes has been associated with adverse prenatal outcomes and deve-lopmental disorders [97]. Research on neonatal umbilical cord blood supported this association by showing that fetuses subjected to their mother’s symptoms of sleep-disturbed breathing during pregnancy had shorter telomere lengths than their counterparts [98]. According to a research, mothers having OSA had, 2.31, 1.34, 1.76 1.74, 1.63, 1.60, and 3.18 times higher chances of having preterm, SGA, LBW, infants, Cesarean section (CS), ges-tational diabetes, preeclampsia, and prenatal hypertension than mothers without OSA [99]. However, research by Kneitel AW *et al*. found that mothers with and without OSA had similar percentages of SGA infants (23 *vs*. 25%, *p* = 1.0). The study found that maternal OSA is associated with slower fetal development in the third trimester, rather than a birth weight below the 10th percentile. In contrast to the women with OSA, pregnant women who utilized PAP therapy did not have higher chances of decreased fetal development, indicating that PAP therapy may be a promising method of improving the health of the fetus [100]. It is interesting to note that some studies have also discovered a connection between maternal OSA and LGA infants. According to a study by Ayana Telerant *et al*., even mild cases of maternal OSA during pregnancy are linked to rapid fetal growth, which manifests in many growth parameters like weight, length, and adiposity. Additionally, it raises the chance of LGA babies [101]. In a similar vein, Brener *et al*. found that offspring of women with mild SDB had improved adiposity acquisition throughout the initial three years of life, an impaired weight-to-length ratio at birth, and rapid catch-up growth. They also discovered a reduced head circumference at birth and a distinctive pattern of head circumference growth throughout the first three years [102]. In addition to the variations in birth weight, OSA is associated with increased risks of preterm deliveries and a variety of congenital disabilities in the fetus. Bourjeily G *et al*. discovered that OSA was associated with a higher incidence of congenital malformations in children (aOR 1.26, 1.11-1.43), with musculoskeletal anomalies having the highest risk (aOR 1.89, 1.16-3.07) after controlling for comorbidities and potential teratogens. Preterm birth was found to be more common among children born to mothers with OSA (31.3% *vs*. 13.0%, *p* < 0.001). Additionally, infants born to women with OSA needed resuscitation with intubation after birth more often (14.4% *vs*. 4.8%, *p* < 0.001 and 0.5% *vs*. 0.1%, *p* < 0.001, respec-tively), experienced a longer hospital stay (8.28 + 14.5 *vs*. 3.97 + 8.63 days, *p* < 0.001), and were more commonly admitted to neonatal critical care or special care units (48) (Table **2**).

According to a study by Tauman *et al*., 64% of infants born to women with SDB had a low social developmental score at 12 months, compared with 25% of infants born to controls (adjusted *p* = .036; odds ratio, 2.6). Additionally, 41.7% of mothers with SDB reported that their infants snored, compared to 7.5% of controls (*p* = 0.004) [103]. Looking forward, various enhancements in healthcare technology can help transform the screening and diagnosis of OSA in pregnancy. Recent advancements, including wearable home sleep apnea testing tools such as the Watch-PAT device, can facilitate remote sleep pattern monitoring without transferring raw data, thereby pre-serving patient privacy, a crucial consideration in maternal healthcare. Federated learning is a privacy-preserving, decentralized approach to data analysis that keeps personal health information on local devices, while still allowing different hospitals, researchers, or devices to work together by sharing only model updates, not the raw data itself. A recent review on federated learning in smart healthcare highlights how Internet of Things (IoT) appli-cations can help clinicians make diagnosis and treatment decisions based on real-time data, rather than relying on periodic checkups. This can help reduce patient hospital visits and improve their quality of life [64, 104].

## CONCLUSION

OSA during pregnancy is a multifactorial condition influenced by anatomical vulnerability and pregnancy-related hormonal variations, resulting in intermittent hypoxia and disrupted sleep, which may negatively impact both the mother and the health of the fetus. The mother is prone to the development of gestational diabetes, pre-eclampsia, hypertensive disorders, cardiomyopathy, and major depressive disorder. For the fetus, maternal OSA is linked to both growth restriction and macrosomia, in-creased risks of preterm birth, congenital anomalies, neurodevelopmental alterations, and greater neonatal morbidity. While various studies have been investigating the link between maternal OSA and the maternal-fetal outcomes, most of these are observational in nature with inconsistent outcomes.

The American Society of Sleep Medicine (AASM) advo-cates for routine screening using tools such as the STOP-BANG Questionnaire, formal polysomnography (PSG), and home-based apnea testing (HSAT). Despite these mea-sures, underdiagnosis remains common due to non-specific symptoms and the lack of validated pregnancy-specific screening tools. CPAP therapy has been shown to mitigate some adverse outcomes, particularly fetal growth restriction and maternal cardiovascular effects, although treatment adherence and pregnancy-specific data remain limited. BiPAP and implantable devices such as hypo-glossal nerve stimulators are other options; however, their use in pregnancy is less documented. Surgical methods may be effective, but they are rarely recommended during pregnancy due to insufficient evidence of their safety and effectiveness. Further well-designed prospective studies are needed to clarify the association between OSA during pregnancy and maternal-fetal outcomes, as well as the efficacy and long-term effects of treatment.

## Figures and Tables

**Fig. (1):**
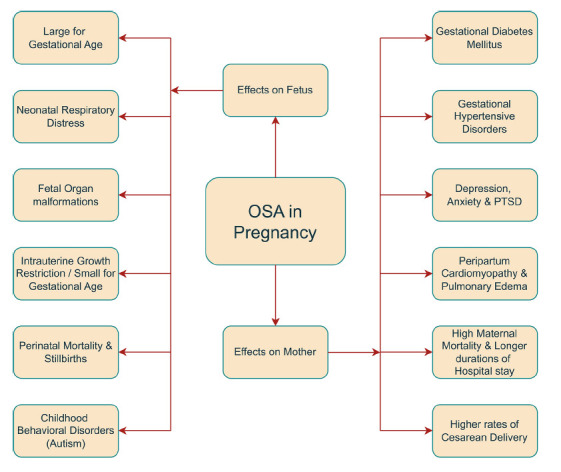
An overview of maternal and fetal outcomes due to OSA in pregnancy.

**Table 1:** Effects on maternal health.

Citation	Results	Conclusion
**Passarella *et al*. (2021) [** **91** **]**	OSA during pregnancy increases the risk of preeclampsia (OR 2.2, 95% CI 2.0-2.4), eclampsia (4.1, 2.4-7.0), chorioamnionitis (1.2-1.8), postpartum hemorrhage (1.2-1.7), venous thromboembolisms (2.7, 2.1-3.4), cesarean births (2.1, 1.9-2.3), and maternal mortality (4.2, 2.2-8.0). Newborns were at high risk of premature births, 1.3 (1.2-1.5), and having congenital abnormalities, 2.3 (1.7-3.0).	OSA in pregnancy is associated with a significantly higher risk of poor maternal and fetal outcomes.
**Liu *et al*. (2018) [****28****]**	A meta-analysis of 33 studies with a total of 3,965 pregnant women found that OSA increases the risk of gestational hypertension (aOR 1.97), gestational diabetes (aOR 1.55), preeclampsia (aOR 2.35), cesarean section (aOR 1.42), and preterm birth (aOR 1.62).	OSA in pregnancy is linked to adverse maternal outcomes. Routine screening and early treatment during late pregnancy are recommended.
**Ryu *et al.* (2023 [** **92** **]**	OSA increased the OR of preeclampsia (OR, 13.1; 95% confidence interval, 1.1-171.3) after adjusting for age, BMI, parity, and abortion history. AHI shows positive correlation with BMI (*r* = 0.515, *P* < 0.001).	OSA is associated with late-onset preeclampsia in 100 overweight Korean pregnant women.
**Reutrakul *et al*. (2013) [** **93** **]**	A case-control study included 15 Non-Pregnant women with normal glucose tolerance (NP-NGT), 15 Pregnant Women with normal glucose tolerance (P-NGT), and 15 pregnant women with gestational diabetes (PGD). Total Sleep Time was lower (median 397 *vs* 464 min, *p* = .02) and AHI was higher (median 8.2 *vs* 2.0, *p* = .05) in P-GDM. Diagnosis of GDM was significantly associated with OSA diagnosis (OR 6.60; 95% CI, 1.15-37.96).	Sleep disturbances are higher in pregnant females with GDM compared to those with normal glucose tolerance.
**Bublitz *et al*. (2018) [** **94** **]**	OSA in gestational diabetes is linked to dysregulation of the hypothalamic-pituitary-adrenal axis, indicated by elevated cortisol levels (r = 0.45, *p* < 0.05). Blunted cortisol awakening responses were observed in females with OSA.	OSA is related to preserved circadian variation and suppressed cortisol awakening responses.
**Yang *et al*. (2020) [****42****]**	A systematic review and meta-analysis of 65 studies found that sleep disturbances, including OSA, were linked to premature deliveries (aOR: 1.95; 95% CI: 1.55-2.45), gestational diabetes mellitus (aOR: 1.96; 95% CI: 1.62-2.38), preeclampsia (aOR: 2.77; 95% CI: 1.81-4.24), cesarean delivery (aOR: 1.99; 95% CI: 1.70-2.33), and depression (aOR: 3.98; 95% CI: 2.74-5.77).	Sleep difficulties during pregnancy were linked to worse perinatal outcomes.
**Lu *et al.* (2021) [** **51** **]**	Sleep disturbances were linked to higher rates of pre-eclampsia (OR 2.80, 95% CI: 2.38-3.30), gestational hypertension (1.74, 1.54-1.97), GDM (1.59, 1.45-1.76), cesarean section (1.47, 1.31-1.64), premature deliveries (1.38, 1.26-1.51), LGA (1.40, 1.11-1.77), and stillbirth (1.25, 1.08-1.45), however, not SGA (1 .03, 0 .92- 1 .16), or LBW (1 .27, 0 .98- 1.64)	This systematic review and meta-analysis of 120 studies, including 58,123,250 pregnant women, found that sleep disturbances during pregnancy were linked to adverse maternal and fetal outcomes.
**Rubio *et al.* (2022) [** **41** **]**	Females at greater risk of OSA were more likely to have antepartum depression (aOR = 2.36; 95% CI: 1.57-3.56), generalized anxiety (aOR = 2.02, 95% CI: 1.36-3.00), and symptoms related to PTSD (aOR = 2.14; 95%CI: 1.43-3.21)	Maternal psychiatric disorders such as depression, anxiety, and PTSD are linked to poor sleep quality and an increased risk of OSA.
**Louis *et al.* (2014) [** **95** **]**	After correcting for obesity and other factors, pregnant females with OSA had higher risks of cardiomyopathy (OR, 9.0; 95% CI, 7.5-10.9), pulmonary embolism (OR, 4.5; 95% CI, 2.3-8.9), and hospital deaths (95% CI, 2.4-11.5).	OSA during pregnancy correlates with increased cardiovascular morbidity and in-hospital mortality.
**Malhamé *et al*. (2022) [** **70** **]**	Preeclamptic females with concomitant OSA had greater cardiovascular morbidity than females without OSA (OR 5.05, 95% CI 2.28-11.17) and increased healthcare utilization (OR 2.26, 95% CI 1.45-3.52).	OSA increases the risk for cardiovascular morbidity and healthcare utilization.
**Lui *et al*. (2021) [** **96** **]**	OSA in pregnancy is associated with longer hospital stays >5 days (aOR 2.42, 95% CI 2.21 to 2.65, but was not linked to increased hospital deaths.	OSA increases maternal morbidity and hospital stay after delivery, but does not increase mortality.

**Table 2:** Effects of OSA on the fetus.

**Ravishankar *et al*. (2015) [** **15****]**	Increased expression of CAIX in the extravillous trophoblast and fetal normoblastemia.	SDB during pregnancy is associated with chronic fetoplacental hypoxia and uteroplacental underperfusion.
**Cànaves-Gómez *et al.* (2024) [****97** **]**	WCB samples from women with OSA showed differential expression of 3258 mRNAs. Five hundred sixty-eight mRNAs were considerably downregulated, while 2690 mRNAs were significantly upregulated. Following maternal OSA, 13 candidate genes were found to be potentially dysregulated in WCB cells.	Maternal OSA strongly impacts fetal gene expression. Negative prenatal outcomes and developmental disorders have been linked to these genes' dysregulation.
**Salihu *et al.* (2015) [****98****]**	Shorter telomere lengths in cord blood DNA.	Fetuses exposed to maternal symptoms of sleep-disordered breathing during pregnancy had shorter telomere lengths.
**Chen *et al.* (2012) [** **99****]**	Mothers with OSA had 1.76 (95% confidence interval [CI], 1.28-2.40), 2.31(95% CI, 1.77-3.01), 1.34 (95% CI, 1.09-1.66), 1.74 (95% CI, 1.48-2.04), 1.60 (95% CI, 2.16-11.26), 1.63, and 3.18 times more chances of having LBW, preterm, small for gestational age (SGA) infants, Cesarean section (CS), preeclampsia, gestational diabetes, and prenatal hypertension than mothers without OSA.	Pregnant women with OSA have a higher chance of having LBW, preterm, and SGA children, CS, and preeclampsia.
**Kneitel *et al*. (2018) [** **100****]**	There were no notable differences in the proportion of children with birth weight <10th centile between women with and without OSA (23 *vs*.25%, *p*=1.0). However, there were significant variations in the proportion of fetal growth slowing in the last trimester of pregnancy (61 *vs*. 29%, *p*=0.0095).	Positive airway pressure is likely to help with the reduced fetal development associated with OSA.
**Telerant *et al.* (2018) [** **101****]**	Rapid fetal growth in the third trimester.	Mild OSA is associated with significantly higher birth weight percentiles, longer gestational age, increased birth length, and thicker triceps in newborns.
**Brener *et al.* (2020) [** **102****]**	Children of mothers with mild SDB had a compromised weight-to-length ratio at birth, rapid catch-up growth, and increased weight status with enhanced adiposity acquisition throughout the first three years of life. Additionally, they had a significantly smaller head circumference at birth (P = 0.004), exhibiting a distinctive pattern of catch-up growth by the end of the first year of life (*P* = 0.018).	The intrauterine environment of a mother with mild SDB affects growth and adiposity acquisition, along with the expansion of head circumference during the first three years of life.
**Bourjeily *et al.* (2019) [** **48****]**	Higher risk of congenital anomalies in offspring (aOR 1.26, 1.11 to 1.43), with the highest risk of musculoskeletal anomalies (aOR 1.89, 1.16 to 3.07). Preterm birth was found to be more common (31.3% *vs*. 13.0%, *p* < 0.001), also these infants needed longer hospital stays (8.28 + 14.5 *vs*. 3.97 + 8.63 days, *p* < 0.001).	OSA is linked to several congenital anomalies; preterm birth, birth complications, and longer hospital stays.
**Tauman *et al.* (2015) [** **103****]**	Sixty-four % of infants born to mothers with SDB had a low social developmental score at 12 months (adjusted *p* = .036; odds ratio, 16.7). Moreover, 41.7% of mothers with SDB reported infant snoring compared to 7.5% of controls. (*p* = 0.004).	Maternal SDB during pregnancy may affect social development at 1 year and is also related to higher chances of snoring in infants.

## LIST OF ABBREVIATIONS

OSA: Obstructive Sleep Apnea
IUGR: Intrauterine Growth Retardation
PAP: Positive Airway Pressure
CPAP: Continuous Positive Airway Pressure
BiPAP: Bilevel Positive Airway Pressure
AASM: American Academy of Sleep Medicine
HDP: Hypertensive Disorders of Pregnancy
BMI: Body Mass Index
hCG: Human Chorionic Gonadotropin
hPL: Human Placental Lactogen
ROS: Reactive Oxygen Species
NFKB: Nuclear Factor Kappa B
TNF-alpha: Tissue Necrosis Factor Alpha
NO: Nitric Oxide
CO_2_: Carbon dioxide
IL: Interleukin
GDM: Gestational Diabetes Mellitus
GAD: Generalized Anxiety Disorder
PTSD: Post Traumatic Stress Disorder
SGA: Small for Gestational Age
LBW: Low Birth Weight
LGA: Large for Gestational Age
NRDS: Neonatal Respiratory Distress Syndrome
PTB: Preterm Birth
REM: Rapid Eye Movement
NREM: Non Rapid Eye Movement
SDB: Sleep Disordered Breathing
EPDS: Edinburgh Postnatal Depression Scale
ESS: Epworth Sleepiness Scale
BATE: BMI and Tongue enlargement
PSG: Polysomnography
AHI: Apnea Hypopnea Index
RDI: Respiratory Disturbance Index
REI: Respiratory Event Index
RERAs: Respiratory Effort-related Arousals
HST: Home Sleep Test
ODI: Oxygen Desaturation Index
HGNS: Hypoglossal Nerve Stimulation
MAD: Mandibular Advancement Devices
MMA: Maxillomandibular Advancement Surgery
NIS: National Inpatient Sample
CAIX: Carbonic Anhydrase IX
WCB: Womb cell Samples
CS: Cesarean Section
